# Is Teaching Less Challenging for Career Switchers? First and Second Career Teachers’ Appraisal of Professional Challenges and Their Intention to Leave Teaching

**DOI:** 10.3389/fpsyg.2019.03067

**Published:** 2020-01-22

**Authors:** Larissa Maria Troesch, Catherine Eve Bauer

**Affiliations:** Teachers Biography and Professionalization, Institute for Research, Development and Evaluation, Berne University of Teacher Education, Bern, Switzerland

**Keywords:** second career teachers, professional challenges, job demands, teacher attrition, intention to leave the profession

## Abstract

Teacher attrition is a major problem in many countries. One possible and widely spread counter measure is to recruit persons from other occupational fields to become teachers. Although the existent literature suggests that second career teachers (SCT) have additional resources compared to first career teachers (FCT), empirical data are still scarce on whether SCT are able to transfer prior skills and knowledge into teaching, how this affects the way they deal with professional demands, as well as their intentions to stay in their new profession. On this basis, the present study explores whether FCT and SCT differ in how challenged they feel by typical professional demands associated with teaching, and in what way their challenge appraisals are relevant for the intention to leave the profession. A questionnaire survey was conducted with a sample of 297 teachers, comprising 193 FCT and 104 SCT. Both groups had had regular teacher training, resulting in a full teacher diploma. Professional demands associated with student learning and assessment were rated as most challenging, whereas classroom management, establishing a professional role and cooperation with colleagues were perceived as less challenging. A group difference was found in professional demands concerning student learning and assessment, where SCT felt less challenged than FCT. Also, SCT were more intent to stay in the teaching profession. Further analyses showed that both group differences are mainly attributable to the higher proportion of male teachers among SCT, as well as to their higher general self-efficacy beliefs. Both career background and the degree to which the teachers felt challenged in their job played a subordinate role for the intention to leave the teaching profession. The findings indicate that SCTs’ background as career switchers might be less important for coping with specific professional demands than the existent research literature implies. On the other hand, they indicate that SCT feel nearly as challenged when starting to teach as traditional teachers, and may have the same needs for good teacher induction.

## Introduction

Teaching is considered a highly demanding occupation, as it involves a broad range of job demands that call for multifaceted skills and a high degree of flexibility (e.g., Frey, 2014; Keller-Schneider et al., 2018). While student learning and achievement is often seen as a teacher’s core business, being a teacher involves many other sometimes underestimated tasks such as school development, cooperation within and outside the school, or administrative tasks (Danielson, 2013; Frey, 2014). These professional tasks and related professional demands are potential stressors as defined by the transactional stress theory (Lazarus and Folkman, 1984). Teacher stress is widely recognized as a primary factor in causing low job wellbeing (Skaalvik and Skaalvik, 2015) as well as turnover and attrition (McCarthy et al., 2016). General protective and risk factors for teacher attrition have been well documented (cf. Kyriacou, 2001; Montgomery and Rupp, 2005; Borman and Dowling, 2008; Schaefer, 2013), and in the last decade, research on the association between the perception of selected job demands and the intention to quit teaching has been intensified as well (Skaalvik and Skaalvik, 2011, 2018). These questions are of particular interest also for second career teachers (SCT). In contrast to first career teachers (FCT), who pursue teaching as a first career after completing their high school diploma, SCT have completed at least one professional training prior to becoming teachers. In many countries, considerable resources are being invested in the recruitment and training of SCT, often as a measure to compensate for teacher shortages; yet, there is only scarce information concerning SCTs’ ability to cope with the demands of their new profession, and their long-term engagement in teaching. The existent literature implies that SCT bring skills and resources into the teaching profession that are highly relevant for coping with teachers’ professional demands (e.g., Tigchelaar et al., 2010). But do SCT actually appraise professional demands differently from FCT, and in what way are their appraisals connected to their intentions to stay in the profession or leave teaching again? The present article explores these questions.

## Theoretical Framework

### Professional Demands as Challenges

Job demands are defined as the physical, social, or organizational aspects of an individual’s work activities that require effort and are thus associated with physiological and/or psychological costs (Demerouti and Bakker, 2011). This definition includes both general demands that are not bound to a particular profession, i.e., demands that can occur in every job, such as work overload or interpersonal conflict, as well as profession-specific demands (hereafter referred to as “professional demands”), i.e., the requirement to cope with the profession’s typical work tasks, calling for specific resources in the form of professional knowledge and skills. Teaching-related professional demands require teachers to activate a broad range of adequate resources; this can lead to feelings of stress, especially in early career teachers (Voss and Kunter, 2019). On the other hand, professional demands can be important sources of professional development, as the gap between situational requirements and an individual’s resources, if not too wide, fosters the acquisition of new skills and resources (Keller-Schneider et al., 2018). Other than the exposure to general job demands such as work conflict, the mastery of professional demands that are specific to the teaching profession – demands related to classroom management or parent conferences, for instance – will inevitably contribute to the expansion or consolidation of a teacher’s professional competencies. In this sense, teaching-related professional demands always imply a potential gain in the form of professional growth and personal achievement, making them challenges in the sense of the transactional stress model by Lazarus and Folkman (1984) or, as LePine et al. (2005) suggest, challenge stressors as opposed to hindrance stressors.

The transactional stress model (Lazarus and Folkman, 1984) puts appraisals at the heart of the stress process and considers the individual’s personal resources. The model explains feelings of stress as the product of a complex and highly subjective transaction between situational demands and individual resources that involves two major appraisal processes: Primary appraisal, evaluating a given situation as either irrelevant, positive or stressful, and secondary appraisal, evaluating coping options and resources. The primary appraisal process leads to stressful appraisals if the situation’s demands tax or exceed the individual’s resources. A stressful appraisal can occur in the form of harm or loss, if some sort of physical or emotional damage has already occurred; in the form of threat, if the situation exceeds the person’s resources, and bears the potential of future damage; or in the form of challenge, if the situation, although taxing the person’s resources, bears the potential of success and an expansion of resources. The boundaries between the three forms of stressors are fluid: e.g., a challenge, albeit potentially positive, can turn into a negative stressor if the second appraisal exposes the available resources as insufficient.

Of the three suggested stressors, challenge has probably been the least studied so far. As Smith and Kirby (2011) point out, the transactional model’s definition of stress has often been interpreted rather restrictively, limiting its occurrence to situations in which the demands clearly exceed the resources, precluding challenge-related forms of stress. This interpretation disregards the crucial role of appraisal in the stress process, and the fact that an individual’s assessment of a situation as either threatening or challenging is context-dependent. Indeed, current stress research – particularly the widely used job demands-resources framework – regards job demands primarily as negative stressors, and considers appraisal processes rather rarely (e.g., Bakker and Demerouti, 2017). Yet, some authors have chosen a different approach. As mentioned above, LePine and colleagues suggest a differentiation between hindrance and challenge job demands (LePine et al., 2005), defining hindrance job demands as negative stressors that interfere with an individual’s goals, and challenge job demands as positive stressors that can promote growth and achievement. Empirical findings corroborate this approach: As predicted by the transactional model, challenge appraisals mediate the relationship between stressors and outcomes such as job satisfaction or turnover intentions (Webster et al., 2011; Searle and Auton, 2014), but are fluid in the sense that stressors can be simultaneously appraised as both a challenge and hindrance (Webster et al., 2011). These studies provide evidence that challenge appraisals might be an important link in the relationship between job demands and work outcomes such as feelings of stress or turnover intention. In order to look at teachers’ challenge appraisals and their role for turnover intentions, it is necessary to first establish what professional demands are specific to the teaching profession.

### Teachers’ Professional Demands

Many international studies have identified a wide range of teacher job demands over the past decades (e.g., Kyriacou, 2001; Gu and Day, 2007; Keller-Schneider, 2014). Among the profession-specific demands that are perceived as most challenging are discipline problems, low student motivation, ill-defined roles between members of the teaching staff, difficulties in teamwork and great diversity of the student body (Hakanen et al., 2006; Keller-Schneider, 2010; Skaalvik and Skaalvik, 2018). In the first few years of their careers in particular, mastering job-related tasks places high demands on teachers (Keller-Schneider, 2014). In order to be able to examine the ways how teachers appraise specific professional demands and the way they match their professional skills, Keller-Schneider developed an empirically grounded model of the perception of professional demands (Keller-Schneider, 2008; Keller-Schneider et al., 2018). Based on interviews with school teachers and supervisors, and pursuing a factor analytic approach, Keller-Schneider found that the professional demands teachers experience in their daily work can be allocated to four major domains (Keller-Schneider et al., 2018):

- **Teaching to Meet Individual Students’ Needs (“Teaching”):** Tasks and demands that are directly linked to student learning and assessment, i.e., the need to choose instructional approaches and methods that cover a wide range of student abilities, interests, and individual needs, including assessment and cooperation with parents.
- **Professional Role and Identity (“Professional Role”):** Tasks and demands related to the need to develop a professional identity, and to establish performance standards for the individual teacher’s own work.
- **Adaptive Classroom Management (“Classroom Management”):** Tasks and demands associated with the need to find effective ways to lead and guide student groups and to establish engaging and healthy learning environments, including lesson structuring and handling of group dynamics.
- **Co-constructive Cooperation Within the School (“Cooperation”):** Tasks and demands linked to a teacher’s interactions and functions as professional member of the school community, including collaboration with colleagues, school principals, and other professionals in the school system.

Following the transactional stress model outlined above, Keller-Schneider’s approach suggests that it is not a professional demand *per se*, but the affected person’s subjective appraisal that is crucial for the individual stress response. An empirical evaluation of the model showed that of these professional demands, cooperation was appraised as the least challenging by beginning teachers, whereas teaching, professional role and identity and adaptive classroom management were rated as more challenging (Keller-Schneider et al., 2018). To what extent teachers felt challenged varied with their career phase: Beginning teachers felt more challenged by the demand to establish their professional role and identity as a teacher, but less challenged by cooperation tasks than more experienced teachers, suggesting that they perceived cooperation with their colleagues more as a resource than a challenge.

### The Role of Professional Demands for Teacher Attrition

Teachers often do not remain in the teaching profession for their entire working life. International findings on teacher attrition and retention are difficult to compare because they often measure different things. However, there is agreement that many countries are struggling to train and retain a sufficient number of qualified teachers, and that the rate of leaving is particularly high among young teachers (e.g., OECD, 2005; Clandinin et al., 2015).

The significance of work-related demands for teacher attrition becomes clear in surveys on the reasons for leaving the profession: Among the most frequently mentioned reasons for attrition are high demands and job stress in general, work overload, the desire for better career options, family responsibilities and lack of administrative support (Kersaint et al., 2007; Druschke and Seibt, 2016). Skaalvik and Skaalvik have shown that workload and time pressure are among the job demands that are most strongly related to the intention to leave teaching, mediated by low job well-being (Skaalvik and Skaalvik, 2011, 2018). These findings highlight the impact of general job demands for teacher attrition. Yet, they explain little whether the many professional demands that are specific to the teaching profession contribute differently to the intention to leave teaching again. Is stress related to classroom management more influential than stress related to cooperation, for instance?

This is a complex question since, as explained above, work-related demands can be hindrance stressors and challenge stressors at the same time (Webster et al., 2011). The teaching profession indeed has the reputation of being both highly demanding and highly rewarding (Johnson and Birkeland, 2003; Skaalvik and Skaalvik, 2015). Teachers’ challenge appraisals might be a crucial factor to explain this supposed discrepancy, and how it is related to teacher attrition, as professional challenges imply potential rewards on the one hand, but are on the other hand a form of stress in the sense of the transactional model (LePine et al., 2005). Teachers’ appraisals of work-related demands are influenced by personal resources such as professional skills, self-efficacy beliefs, goals and expectations (Kyriacou and Sutcliffe, 1978; Rudow, 1999; Troesch and Bauer, 2017a), and in turn have an influence on job-wellbeing and eventually the intention to stay in the profession (Skaalvik and Skaalvik, 2011). The relevance of personal resources for the appraisal process indicates that teachers’ individual biographies might have a substantial impact on how they perceive professional challenges, and whether they intend to stay in teaching. A newer discourse, framing teacher attrition as an identity-making process, points into a similar direction (Schaefer et al., 2012). Emphasizing the significance of personal goals and experiences, these authors argue that leaving the profession can be a deliberate life-making process if teachers are strongly challenged by their professional role and identity, and experience a frustrating discrepancy between their original images of what kind of a teacher they wanted to be, and their actual experience as teachers.

Against this background, we suggest that a focus on teachers’ biographies is important to gain a deeper understanding of how challenging specific professional demands are for different groups of teachers, and how challenge appraisals are connected to the intention to stay in the profession, or to leave teaching again.

### Second Career Teachers (SCT): Characteristics and Resources

In the last decades, the development of alternative routes into teaching has expanded considerably in many countries, often combining academic curricula with work-place learning in order to efficiently qualify professionals for the teaching profession (e.g., Tigchelaar et al., 2010; Marinell and Johnson, 2014). As a measure to compensate for teacher shortages, these programs offer a way to expand the workforce by tapping a pool of professionals from other fields. As conditions, standards and training modalities for career changers vary widely between countries and even universities, SCT are a very heterogeneous group of teachers in terms of age, prior educational and professional background, and duration as well as content of teacher training. Yet, there are few common characteristics that will be addressed in the following paragraphs.

#### Life Experience and Professional Expertise

Having completed at least one professional qualification prior to switching to teaching, SCT are older than FCT, with the consequence that they find themselves in a different phase of their lives, and are more likely to have children of their own (e.g., Tigchelaar et al., 2010; Troesch and Bauer, 2017a). From a career development perspective, SCT have at least once experienced the novice phase of career entry already, and reached a certain level of expertise in another domain, making them “expert novices” when entering the teaching profession (Williams, 2013). Yet, despite the widespread practice of recruiting professionals to become teachers, it is not clear whether SCT are actually able to benefit from their prior training and work experience when having to deal with teacher professional demands. Findings of expertise research generally emphasize that expertise is domain-specific and not easily transferable (Gruber and Mandl, 1996). The accumulation of work and life experiences does not automatically lead to better teaching skills, but can only translate into a professional repertoire if reflected upon and purposefully implemented in the classroom (Freidus and Krasnow, 1991; Mayotte, 2003). Established knowledge and routines can even interfere with professional development in the new profession (Bauer et al., 2017).

#### Biographical Agency and Self-Efficacy

It is a basic assumption of life-course research that important personal resources, such as work-related knowledge and control beliefs, are not only shaped by curricular approaches, but also by the individual occupational biography (Moen, 2003; Heinz et al., 2004). Life-course transitions such as career entry and development, including career changes, can be understood as processes of self-socialization, built upon the dynamics of individual agency in varying social contexts over time, when individuals tackle challenges, pursue their aspirations and try to cope with disappointment by selecting pathways and solutions that they perceive as promising. In the context of these processes, experiences related to work options and conditions are translated into biographical action orientations, which are part of a strategic adaptation in order to regain a sense of control, identity and contingence, and which shape further decision-making.

Sense of control is an important personal resource that is associated with a higher chance to move into other occupations or reentering the educational system (Moen, 2003). Starting from this premise, it should be assumed that SCT do not only have a headstart concerning age, but also concerning their biographical agency, and ultimately their control beliefs. Research on SCTs’ self-efficacy beliefs corroborates this theoretical assumption. Indeed, SCT have been shown to have self-efficacy beliefs above average compared to teachers in general (Weinmann-Lutz et al., 2006). Self-efficacy beliefs are defined as an individual’s conviction about his or her capabilities to accomplish a task when faced with a challenge (Bandura, 1997). Mastery experiences are thought to be one of the most important sources of self-efficacy beliefs. Our own data corroborate that self-efficacy beliefs are higher in SCT than in FCT, but also suggest that they have a higher impact on job stress in SCT (Troesch and Bauer, 2017a), making self-efficacy a crucial control variable in the investigation of professional demands and work outcomes in SCT.

#### Other Characteristics

Other studies suggest that there are even more desirable qualifications and skills associated with career change into teaching, such as pronounced intrinsic motivations to teach (Williams and Forgasz, 2009; Zuzovsky and Donitsa-Schmidt, 2014), high empathy and communication skills (Freidus and Krasnow, 1991), and a great interest in further education and professional development (Weinmann-Lutz et al., 2006; for an overview, see Tigchelaar et al., 2010). On these grounds, it seems safe to assume that SCT bring additional resources into the teaching profession that might facilitate the ability to cope with professional demands, leading to less teacher stress in SCT compared to FCT. Up to now, there is only very limited research concerning this assumption. Keller-Schneider et al. (2016) found no significant differences between SCT and FCT regarding the extent to which they felt challenged by typical professional demands. However, the SCT in Keller-Schneider’s study alternatively certified; it seems plausible that potential benefits of prior career experiences had been outweighed by the shortened teacher training. Our own data revealed no significant differences between SCT and FCT concerning job stress either, but SCT were more satisfied with their jobs than FCT (Troesch and Bauer, 2017a).

Concerning SCTs’ long-term retention in the teaching profession, the scarce empirical data is conflicting: While some authors suggest that SCT might be particularly prone to switching jobs or even careers again if working conditions do not meet their standards (Johnson and Birkeland, 2003), other studies show higher intentions to leave the profession in FCT than SCT (Troesch and Bauer, 2017b) or no group differences at all (Boyd et al., 2011a; Kocher et al., 2019). The inconsistent nature of these findings might be due to the heterogeneous samples of SCT involved in the different studies, particularly concerning their prior careers and qualifications, but also their qualifications as teachers. Moreover, there is evidence that the nature of teacher training as well as the previous educational background has an influence on retention (Zuzovsky and Donitsa-Schmidt, 2017). In order to learn more about whether a previous career background *per se* has any influence on teacher attrition, it would be necessary to compare SCT and FCT with an identical or similar teacher training background.

### Research Questions

The literature reviewed above shows that due to their career pathways, SCT might have knowledge and resources that facilitate the mastery of professional demands, and reduce attrition from teaching (Tigchelaar et al., 2010). However, there is little empirical data concerning SCTs’ actual appraisal of professional demands and how they influence their turnover intentions. Drawing on the transactional stress model (Lazarus and Folkman, 1984), professional demands can be conceptualized as challenges, i.e., a type of stressor that can have positive consequences in terms of personal and professional growth, but that can turn into a negative stressor if the challenge is too high and exceeding the individual’s resources. As work-related demands and feelings of stress are strong predictors for attrition, it can be assumed that teachers’ challenge appraisals of their daily professional tasks are an important influencing factor for their intentions to stay in the profession. However, it is unclear whether SCT and FCT react differently to the specific demands in the teacher profession, and whether different professional demands have a different impact on the intention to leave the profession. In order to address these issues, the present study addresses the following research questions:

1. Challenge appraisals: Do SCT and FCT differ regarding how challenged they feel by typical professional demands?
2. Intention to leave teaching: Do SCT and FCT differ regarding their intentions to leave the teaching profession?
3. Relationship between challenge appraisals and intention to leave the profession:
  a. Does the relationship between challenge appraisals and intention to leave vary for different professional demands?
  b. Do SCT and FCT differ regarding the relationship between challenge appraisals and intention to leave?

## Materials and Methods

### Participants

The data are based on the project “Professionals as Teachers,” a Swiss research project investigating first and SCT’ job well-being and retention (Bauer and Hostettler, 2012; Troesch and Bauer, 2017a). All teachers belonging to the 2004–2007 graduation cohorts of the Berne University of Teacher Education (Switzerland) were contacted by questionnaire (912 persons) 7–10 years after graduation, of which 400 took part in the study. The aim of the present study was to examine challenge appraisals in the teaching profession. This appraisal requires current experience in teaching. Therefore, the present analyses do not include persons who were not working as teachers at the time of the survey. This led to a sub-sample of 297 persons who were still working in the teaching profession at that time (average age = 34.21; *SD* = 6.54; 229 women). Participants who had completed a prior vocational training before entering teacher training, and had thus pursued a prior career, were allocated to the SCT group; this definition applied to 104 persons. Participants who had completed teacher training as a first vocational training were allocated to the FCT group; this definition applied to 193 participants. Although most SCT had entered teacher training via a specific admission for SCT, including a preparatory course and entrance exam, all participants had completed regular teacher training either for kindergarten, primary or secondary education. In the SCT group, the average amount of work experience in their previous career was 7.14 years (*SD* = 7.87). Approximately one third of the SCT had worked in administrative professions prior to teacher training; in the other two thirds, most had had prior careers in health and craft occupations. Sample descriptives are shown in Tables 1, 2.

**TABLE 1:** Descriptive statistics of study variables.

Variable	Min	Max	Total sample		SCT		FCT		Group comparison^1^			
			*M (%)*	*SD*	*M (%)*	*SD*	*M (%)*	*SD*	*t*	*df*	*p*	*Hedge’s g*
Percentage female	0	100	(77.10)	−	(62.50)	−	(84.97)	−	19.34	1	< 0.001	−
Age	28	61	34.21	6.54	39.10	8.47	31.58	2.74	–8.79	115	< 0.001	–1.37
Years experience	0	14	7.62	1.57	7.46	1.84	7.70	1.40	1.17	169	0.24	0.15
% Employment	0	110	73.00	25.56	74.02	24.21	72.45	26.30	–0.50	295	0.62	–0.06
**Self-efficacy**												
General	10	40	31.09	3.67	31.67	3.64	30.78	3.66	–2.00	295	< 0.05	–0.24
Teacher	10	40	30.63	3.41	30.96	3.57	30.45	3.31	–1.23	295	0.22	–0.15
**Challenge appraisals of professional demands**												
Teaching	1	6	4.33	0.95	4.11	0.93	4.45	0.94	2.96	295	< 0.01	0.36
Class. management	1	6	3.97	0.98	3.91	0.99	4.00	0.98	0.72	295	0.47	0.09
Cooperation	1	6	2.86	1.04	2.87	1.05	2.83	1.03	0.31	295	0.76	–0.04
Professional role	1	6	3.88	0.95	3.75	0.96	3.94	0.94	1.66	295	0.10	0.20
Intention to leave^2^	1	4	−	−	134.67^2^		156.72^2^	−	–2.27	−	< 0.05	0.13

*SCT = second career teachers (*n* = 104); FCT = first career teachers (*n* = 193); *M* = Mean; Class. Management = classroom management. ^1^*X*^2^ – statistics for nominal data (percentage female); Independent Samples Mann–Whitney *U*-Test/*z* – statistics and *r* for ordinal data (intention to leave); ^2^Instead of means, mean ranks are reported, median was 2 for the total sample, as well as for SCT and FCT; Analyses with imputed data.*

**TABLE 2:** Correlation of study variables.

Variable	1	2	3	4	5	6	7	8	9	10
(1) Gender	−									
(2) Age	–0.17^∗∗^									
(3) Years experience	–0.00	0.11								
(4) % Employment	–0.17^∗∗^	–0.02	–0.05							
**Self-efficacy**										
(5) General	–0.01	0.04	0.00	0.17^∗∗^						
(6) Teacher	–0.01	–0.01	–0.07	0.16^∗∗^	0.50^∗∗∗^					
**Challenge appraisals of professional demands**										
(7) Teaching	0.21^∗∗∗^	–0.17^∗∗^	0.07	0.03	–0.15^∗∗^	–0.11				
(8) Class. management	0.09	0.01	0.03	–0.03	–0.19^∗∗^	–0.26^∗∗∗^	0.59^∗∗∗^			
(9) Cooperation	0.06	0.05	–0.01	0.03	–0.19^∗∗^	–0.18^∗∗^	0.42^∗∗∗^	0.54^∗∗∗^		
(10) Professional role	0.05	–0.05	–0.01	0.05	–0.25^∗∗∗^	–0.18^∗∗^	0.61^∗∗∗^	0.69^∗∗∗^	0.57^∗∗∗^	
(11) Intention to leave	–0.16^∗∗^	–0.03	0.08	–0.02	–0.20^∗∗^	–0.19^∗∗^	–0.06	–0.06	–0.03	–0.03

*Gender: 0 = male, 1 = female; Class. Management = classroom management. Analyses with imputed data. *N* = 297. ^∗∗^*p* < 0.01; ^∗∗∗^*p* < 0.001.*

### Instruments

#### Challenge Appraisal of Professional Demands

The appraisal of challenge when confronted with specific professional demands were measured using the short scale of Keller-Schneiders’ Professional Requirement Scales (EABest-k; Keller-Schneider, 2010, 2014). For this purpose, the respondents were asked to assess 25 typical teacher professional demands regarding the extent to which they perceived them as challenging (“beanspruchend”; 1 = little to 6 = very much). The full scale is available in Supplementary Material 1. To analyze the structure of the typical professional demands, Keller-Schneider (2010) conducted a factor analysis revealing four factors: teaching to meet individual students’ needs (hereafter referred to as “teaching”; 8 items, e.g., “implement individualized instruction in the classroom”), adaptive classroom management (hereafter referred to as “classroom management”; 6 items, e.g., “Be aware of and lead classroom dynamics”), cooperation within the school (hereafter referred to as “cooperation”; 4 items, e.g., “develop a successful cooperation with the principle”) and professional role and identity (hereafter referred to as “professional role”; 7 items, e.g., “taking care of ongoing development”). The mean value of the corresponding items was used to form the subscales. The internal consistencies of the subscales of the present study can be classified as good with α = 0.84 for teaching, α = 0.82 for classroom management, α = 0.73 for cooperation and α = 0.81 for professional role.

#### Intention to Leave the Profession

The intention to leave the teaching profession was measured with a single question: “Can you imagine leaving teaching in the foreseeable future?” Answering options were 1 = no, 2 = rather no, 3 = rather yes, and 4 = yes. This means that a higher value corresponds to a higher intention to leave.

#### General and Teacher Self-Efficacy

General self-efficacy was assessed with the respective instrument by Schwarzer and Jerusalem (1999), and teacher self-efficacy with the respective instrument by Schwarzer and Schmitz (1999). Both scales contain 10 items which had to be rated on a scale from 1 = absolutely do not agree to 4 = “I absolutely agree” (Supplementary Material 1). Examples for general self-efficacy are “If someone opposes me, I can find the means and ways to get what I want” and “I can remain calm when facing difficulties because I can rely on my coping abilities.” Examples of teacher self-efficacy expectations are “I know that I can maintain a positive relationship with parents, even when tensions arise” and “I know that I can motivate my students to participate in innovative projects.” The internal consistencies of the scales can be described as very good with α = 0.84 for general self-efficacy and α = 0.74 for teacher self-efficacy. As recommended by Schwarzer and Jerusalem (1999) and Schwarzer and Schmitz (1999), all the item scores were summed up to form the total score with a possible range of 10 to 40.

### Analytic Approach

To test for research question 1, multivariate analyses were calculated with the challenges by professional demands as dependent variables and career path (SCT/FCT), age, gender, number of years of teaching experience, degree of employment as a teacher in percent and self-efficacy (general and teacher self-efficacy) as independent variables. For research questions 2 and 3a, an ordinal regression was calculated to intention to leave the profession. In Model 1, the following independent variables were introduced: age, gender, number of years of teaching experience, degree of employment as a teacher in percent, career path (SCT/FCT) and self-efficacy (general and teacher self-efficacy). In Model 2, the challenge appraisals of the four professional demands were introduced. For research question 3b, ordinal regression analyses were calculated separately for SCT and FCT. In Model 1, age, gender, number of years of teaching experience, degree of employment as a teacher in percent and self-efficacy (general and teacher self-efficacy) were used as independent variables and intention to leave the profession as dependent variable. In Model 2, challenge appraisals of the four professional demands were added.

#### Missing Values

8.08% of the participants had missing on construct-level. One possibility to deal with missing values is multiple imputation. Multiple imputation is regarded as superior to the traditional way of dealing with missing (listwise deletion; pairwise deletion; Graham, 2009). To conduct multiple imputations, we followed the recommendations by Graham et al. (2007) and Newman (2014). Forty datasets were estimated.

## Results

### Research Question 1: Group Differences Regarding Challenge Appraisals

The descriptive analyses show that SCT reported the highest challenges in relation to teaching tasks in the narrower sense, which were appraised as rather challenging. Cooperation with other professionals was appraised as the least challenging, with classroom management and professional role ranging in between (Table 1). As the repeated measures ANOVA^1^ showed, SCT rated teaching as significantly more challenging than cooperation with other professionals (*Mean Difference* = 1.28, *SE* = 0.10, *p* < 0.001) and professional role (*Mean Difference* = 0.36, *SE* = 0.08, *p* < 0.001), but not more challenging than classroom management (*Mean Difference* = 0.20, *SE* = 0.08, *p* = 0.10). However, there was no significant difference in challenge appraisals between classroom management and professional role in SCT (*Mean Difference* = 0.16, *SE* = 0.07, *p* = 0.19). Cooperation with other professionals was rated as less challenging than all other job demands (all > 0.001). A similar picture emerged for FCT: teaching was rated as most challenging and significantly more challenging than all other job demands (all > 0.001). Also, cooperation with other professionals was rated as less challenging compared to all other job demands in FCT (all > 0.001). However, no significant difference was found between challenge appraisals in classroom management and professional role (*Mean Difference* = 0.06, *SE* = 0.06, *p* = 1.00). When analyzing the total sample (SCT and FCT), the job demand teaching was rated as most challenging and significantly more challenging than all other job demands (all > 0.001). Moreover, there was no significant difference in challenge appraisals between classroom management and professional role in the total sample (*Mean Difference* = 0.09, *SE* = 0.05, *p* = 0.24). Cooperation with other professionals was rated as less challenging than all other job demands (all > 0.001).

As *t*-tests revealed, significant group differences between SCT and FCT were only found concerning teaching tasks, where FCT felt significantly more challenged than SCT (Table 1). No significant differences between SCT and FCT were found concerning classroom management, cooperation and professional role.

Multivariate analyses revealed that taking important variables into account, the association between career path (SCT/FCT) and challenge appraisals was not significant (*F*(4,286) = 0.23, *p* = 0.92, η^2^_partial_ = 0.00). When the challenge appraisals were treated separately for different professional demands, career path had no effect for any of them (all < 0.05). However, general self-efficacy (*F*(4,286) = 3.09, *p* < 0.05, η^2^_partial_ = 0.04) and teacher self-efficacy (*F*(4,284) = 3.37, *p* < 0.05, η^2^_partial_ = 0.05) were negatively related to challenge appraisals; gender was positively related to challenge appraisals (*F*(4,286) = 3.33, *p* < 0.05, η^2^_partial_ = 0.04). As illustrated in Table 2, gender was particularly important for challenge appraisal in teaching, with women feeling more challenged by teaching than men (*F*(1,289) = 11.24, *p* < 0.01, η^2^_partial_ = 0.04). Age, years of experience and work-time percentage as a teacher, on the other hand, had no significant effect on challenge appraisals (all > 0.05).

### Research Question 2: Group Differences Regarding Intentions to Leave Teaching

An Independent Samples Mann–Whitney *U*-Test revealed a significant group difference: FCT showed higher intentions to leave teaching compared to SCT (see Table 1). The group effect is small, with both groups expressing a relatively high mean intention to stay in the profession.

Taking important control variables into account, the difference between SCT and FCT in intention to leave the profession disappeared (Table 3). In the first ordinal regression analysis (Model 1), gender as well as general and teacher self-efficacy proved to be significant predictors of the intention to leave the teaching profession. Men were more likely to consider leaving the profession than women. Teachers with lower general and teacher self-efficacy reported higher intentions to leave. Age, number of years of teaching experience and degree of employment as a teacher were not significant for the intention to leave the profession. Model 1 explained 13% of the variance.

**TABLE 3:** Ordinal regression analysis predicting intention to leave the teaching profession, total sample (*N* = 297).

Predictor	Model 1				Model 2			
	*B*	*SE*	*p*	OR	*B*	*SE*	*p*	OR
Gender	–1.06	0.27	< 0.001	2.88	–0.98	0.28	< 0.001	2.66
Age	–0.04	0.02	0.08	0.96	–0.04	0.02	0.07	0.96
Years experience	0.07	0.07	0.35	1.07	0.08	0.07	0.27	1.09
% Employment	0.00	0.00	0.87	1.00	–0.01	0.00	0.85	1.00
Career path	–0.38	0.28	0.18	1.47	–0.38	0.28	0.19	1.46
**Self-efficacy**								
General	–0.07	0.03	< 0.05	0.93	–0.07	0.04	0.06	0.94
Teacher	–0.08	0.04	< 0.05	0.92	–0.09	0.04	< 0.05	0.91
**Challenge appraisals of professional demands**								
Teaching					–0.12	0.16	0.43	0.88
Class. management					–0.26	0.17	0.12	0.77
Cooperation					–0.04	0.13	0.75	0.96
Professional role					0.30	0.18	0.10	1.35

*OR = Odds Ratio; Gender: 0 = male, 1 = female; Career path: 0 = first career teachers, 1 = second career teachers; Class. Management = classroom management. Nagelkerke’s *R*^2^ = 0.13 for Model 1; Nagelkerke’s *R*^2^ = 0.15 for Model 2. Analyses with imputed data.*

### Research Question 3: Relationship Between Challenge Appraisals and Intention to Leave

#### Difference Between Professional Demands

As the correlational analyses reveal (Table 2), challenge appraisals of the four different professional demands were not significantly related to the intention to leave the profession. Moreover, the correlational coefficients were in a similar range (−0.03 until −0.06) indicating that there were no differences in the relations between feeling challenged by specific professional demands and the intention to leave the profession.

This result was confirmed by the ordinal regression analysis: As Model 2 shows (Table 3), none of the challenge appraisals of professional demands reached statistical significance taking important controls into account. Again, career path, age, number of years of teaching experience and degree of employment as a teacher were no significant predictors. Moreover, general self-efficacy was not significantly related to intention to leave the profession in Model 2. However, gender and teacher self-efficacy emerged as relevant predictors. In Model 2, 15% of the variance in intention to leave was explained.

#### Group Differences

In a next step, SCT and FCT were analyzed separately (Tables 4, 5). The challenge appraisals for the four professional demands had no significant effect on intention to leave, neither for SCT nor for FCT. In SCT, but not FCT, gender and general self-efficacy played an important role for the intention to leave the profession. In contrast, teacher self-efficacy played a significant role for the intention to leave only in FCT. For both groups, age, number of years of teaching experience and degree of employment as a teacher were not significant for intention to leave the profession, It is also noteworthy that the included variables explained considerably more variance in the SCT than the FCT group (Model 2: SCT: 29%; FCT: 10%).

**TABLE 4:** Ordinal regression analysis predicting intention to leave the teaching profession, second career teachers (*n* = 104).

Predictor	Model 1				Model 2			
	*B*	*SE*	*p*	OR	*B*	*SE*	*p*	OR
Gender	–1.68	0.41	< 0.001	5.34	–1.57	0.43	< 0.001	4.78
Age	–0.05	0.02	0.06	0.96	–0.05	0.03	0.05	0.95
Years experience	0.05	0.11	0.63	1.06	0.05	0.11	0.62	1.06
% Employment	0.00	0.01	0.81	1.00	0.00	0.01	0.68	1.00
**Self-efficacy**								
General	–0.19	0.06	< 0.01	0.83	–0.19	0.07	< 0.01	0.83
Teacher	–0.01	0.06	0.82	0.99	–0.02	0.07	0.75	0.98
**Challenge appraisals of professional demands**								
Teaching					–0.25	0.30	0.41	0.78
Class. management					0.04	0.37	0.90	1.04
Cooperation					–0.20	0.25	0.41	0.82
Professional role					0.33	0.31	0.29	1.39

*OR = Odds Ratio; Gender: 0 = male, 1 = female; Class. Management = classroom management. Nagelkerke’s *R*^2^ = 0.27 for Model 1; Nagelkerke’s *R*^2^ = 0.29 for Model 2. Analyses with imputed data.*

**TABLE 5:** Ordinal regression analysis predicting intention to leave the teaching profession, first career teachers (*n* = 193).

Predictor	Model 1				Model 2			
	*B*	*SE*	*p*	OR	*B*	*SE*	*p*	OR
Gender	–0.38	0.38	0.34	1.46	–0.36	0.39	0.36	1.44
Age	0.08	0.06	0.15	1.08	0.07	0.06	0.19	1.08
Years experience	0.06	0.10	0.58	1.06	0.07	0.10	0.52	1.07
% Employment	0.00	0.01	0.79	1.00	0.00	0.01	0.84	1.00
**Self-efficacy**								
General	–0.02	0.04	0.71	0.98	–0.01	0.04	0.76	0.99
Teacher	–0.12	0.05	< 0.05	0.88	–0.14	0.05	< 0.01	0.87
**Challenge appraisals of professional demands**								
Teaching					–0.01	0.19	0.89	0.99
Class. management					–0.33	0.20	0.12	0.72
Cooperation					–0.03	0.16	0.86	0.97
Professional role					0.36	0.39	0.23	1.32

*OR = Odds Ratio; Gender: 0 = male, 1 = female; Class. Management = classroom management. Nagelkerke’s *R*^2^ = 0.09 for Model 1; Nagelkerke’s *R*^2^ = 0.10 for Model 2. Analyses with imputed data.*

## Discussion

Due to the increasing flexibilization of educational opportunities and occupational biographies, as well as recurring teacher shortages, SCT are a growing teacher group in many countries. Policy makers who create programs to recruit and train SCT rely heavily on the assumption that having had a previous career is associated with transferable skills and knowledge, resulting in alternative and often shortened training programs for SCT; but in fact, empirical data corroborating this hypothesis are scarce (e.g., Marinell and Johnson, 2014). Findings from mainly qualitative, small-scale studies suggest that SCT are indeed able to draw on their prior work experience and knowledge in certain ways (for an overview, see Tigchelaar et al., 2010). However, there is hardly any data addressing the question whether these experiences can be understood as an expanded set of resources that helps to cope with specific professional demands in the teaching profession. On this background, the present study aimed to investigate to what extent second and FCT feel challenged by typical teacher professional demands and in what way these challenge appraisals influence the intention to stay in the teaching profession.

### Do SCT Feel Less Challenged by Teacher-Specific Professional Demands?

The answer to this question is complex: SCT do feel less challenged, but only to a very limited degree, and it seems that this difference is mainly attributable to SCTs’ personal resources as well as to the gender distribution in this subgroup of teachers.

In line with earlier findings (Keller-Schneider et al., 2018), professional demands directly related to student learning and assessment are rated as most challenging compared to the job demands classroom management, professional role and cooperation with other professionals. This finding might be attributable to the fact that teaching and student assessment are often regarded as a teacher’s core business, and demand a great amount of a teacher’s working hours. FCT felt more challenged by these tasks than SCT, but further analyses showed that this group difference vanishes when age, gender, years of experience and work-time percentage as a teacher as well as general and teacher self-efficacy are being controlled for. Of these control variables, only gender and general as well as teacher self-efficacy were significantly related to challenge appraisals of professional demands. This leads us to two possible interpretations: As the percentage of male teachers is higher in SCT, and males tend to report lower levels of challenge in teaching, the group difference regarding challenge appraisals concerning teaching tasks might also be attributable to gender. On the other hand, SCTs’ lower feelings of challenge might be a consequence of their higher general self-efficacy beliefs. This interpretation would be in line with the findings from life-course research, suggesting that life-course transitions such as career changes facilitate the development of a strong sense of control (Heinz et al., 2004).

The finding that establishing a professional role as well as cooperation with other professionals were perceived as equally challenging by both groups, is to some extent surprising. While occupation-specific skills and knowledge from prior fields of work (e.g., in health or administration jobs) may not be transferable to the teaching profession, cross-domain skills should be. Work experiences usually include forms of collaboration, as well as having to establish a flexible professional role toward different interaction partners within the respective work context, i.e., clients, supervisors, colleagues, etc. Moreover, SCT have been shown to integrate more easily into the school organization (Tigchelaar et al., 2008). Therefore, it would have been plausible that establishing a professional role and cooperation with other professionals are appraised as less challenging by SCT. On the other hand, interview data from the same study (Bauer et al., 2017) showed that SCT often struggled with disappointments as they tended to have high expectations regarding team work in their new profession, but also high expectations regarding their own skills and abilities to cope with professional demands. Teaching differs from many occupations – at least in Switzerland – in that teamwork is only an emerging concept, and that there tend to be less guidelines than in other occupational fields in terms of regular feedbacks or performance standards; substantial differences that might foster disappointments and disorientation in SCT. As SCT are a growing teacher subgroup, it might be beneficial for future research to look more closely into the conditions under which SCTs’ prior work experiences can influence the resources that help to establish a professional role as a teacher, as well as cooperation with other professionals in the school environment.

### Do SCT and FCT Differ Regarding Their Intentions to Leave the Profession?

Similar to the results concerning challenge appraisals, our findings show that FCT had higher intentions to leave the teaching profession than SCT, and that this group difference could be attributed mainly to gender and personal resources, as the group difference between FCT and SCT vanished after taking these control variables into account.

Self-efficacy and gender emerged as relevant predictors for the intention to leave the profession. However, the importance of these predictors differed for SCT and FCT: Among SCT, but not FCT, men were more open toward leaving the profession than women. At the same time, teacher self-efficacy was negatively related to the intention to leave in FCT, but not SCT. The importance of self-efficacy beliefs in the workplace for teacher retention has been shown in many studies (for an overview, see Chambers Mack et al., 2019). Our findings corroborate these results, with the addition that for SCT, general self-efficacy is more important regarding their intentions to stay teachers, while for FCT, teacher-related self-efficacy seems to be more relevant. One possible explanation for this finding is that SCTs’ cumulative work experiences are reflected in their general self-efficacy rather than their teacher-related self-efficacy, and that their higher general self-efficacy beliefs compensate for possible weaknesses they feel regarding their efficacy as teachers. However, it might also be attributable to a selective drop-out: teachers with limited resources are likely to have left teaching long ago, resulting in them having been excluded from these analyses that included active teachers only. Thus, it is possible that the rate of teachers who have already left teaching is different for SCT or FCT. In order to investigate the connections between career path, personal resources, feelings of challenge and the intention to leave the profession more thoroughly, a longitudinal study would be needed.

On first glance, it might be surprising that gender has an impact on the intention to leave only in SCT, as earlier studies have shown a higher interest in professional development for male teachers in general (Borman and Dowling, 2008). But while one explanation lies in the higher proportion of males in SCT, it is also highly plausible that this effect has to do with different life phases: With a mean age of 40 for SCT and 32 for FCT, FCT matched the life phase between 30 and 35 when female Swiss teachers show a distinct rise in attrition from teaching due to family reasons (SKBF, 2014), leveling the difference between males and females in this subgroup.

The relationship between work experience prior to teaching and intention to leave the profession has rarely been studied, and the findings are inconsistent. On the one hand, a higher age when entering teaching is associated with a higher probability of remaining in the profession (Borman and Dowling, 2008). Because SCT are usually older than FCT, this might indicate that SCT are more prone to remain in the teaching profession. In addition, career changers have particularly strong motivations to teach (Zuzovsky and Donitsa-Schmidt, 2014), which in turn is an important predictor of career retention (Watt et al., 2014). On the other hand, SCT have higher general self-efficacy (Troesch and Bauer, 2017a), which is associated with a higher probability for further career change (Lent et al., 1994). Moreover, SCT have their first career as a fallback career in case teaching should not be as rewarding as anticipated. The present findings corroborate that SCT might be more intent to stay longer in the teaching profession – at least if they have a regular teacher’s diploma as was the case in the present sample. It has to be considered that the present sample of SCT has a regular, full teacher’s degree as opposed to an alternative certificate. Attrition intentions are linked to a subjective cost-benefit-analysis, and the time and financial investments associated with the completion of often shortened alternative teacher training programs for SCT are comparably low (Weinmann-Lutz, 2007). Indeed, teachers with alternative certificates are more likely to leave the profession again (Chambers Mack et al., 2019). However, the effect of alternative certifications on teacher attrition intention might also depend on the specific curriculum: According to Kocher et al. (2019), retention rates might be associated with the extent to which training on the job is part of the alternative certification program. The degree to which teacher are socialized into their profession seems to play a role for retention and attrition.

### What Is the Role of Teachers’ Challenge Appraisals for Their Intention to Leave the Teaching Profession?

The extent to which the teachers felt challenged by typical professional demands of the teaching profession played a subordinate role for their intention to leave the profession. None of the professional demands was a significant predictor in this regard. This finding is somewhat surprising, as professional challenges – while being associated with beneficial outcomes – are still a type of stressor according to the transactional stress model (Lazarus and Folkman, 1984), and can turn into negative stressors if the challenge is too high. Yet, our findings suggest that professional challenges, even if high, are not associated with the intention to leave the profession. There are several interpretations of this phenomenon:

The extent to which an individual feels challenged by a job demand is an indicator for the extent to which relevant resources have to be considered and made available or, if already at disposal, activated. This process can put high pressure on an individual that might be perceived as momentarily stressful. However, these moments of stress are not the same as the long-term, health-impairing process of work stress that leads to exhaustion and attrition due to burnout, as described by many studies, mainly within the job demands-resources framework (Demerouti et al., 2001). As long as teachers are appreciated by their students and feel that they can accomplish something through their work, i.e., as long as they have a sense of self-efficacy, stress and dissatisfaction remain low (Dicke et al., 2018). Teacher self-efficacy was a relevant predictor for intention to leave the profession in the present analyses, corroborating that self-efficacy beliefs buffer the job demands-strain relationship. From this perspective, our results yield an explanation for the often-cited finding that teachers feel both highly strained and highly satisfied at the same time: The professional demands related to teaching are perceived as moderately to highly challenging – the latter in the case of tasks concerning student learning and assessment –, but not to an extent where the career choice is questioned.

Another possible explanation for the weak association between challenge appraisals and intention to leave the profession might be that the perception of professional demands is context-dependent. Workplace factors such as leadership support, organizational characteristics or school climate have been documented as key factors in teacher attrition throughout the literature (Borman and Dowling, 2008; Kukla-Acevedo, 2009; Van Droogenbroeck and Spruyt, 2016). High levels of challenge might lead to a change of workplace – and not the teaching profession – before excessive demands manifest themselves in forms of chronic stress and exhaustion. This interpretation is supported by the study by Boyd et al. (2011b) which showed that professional demands are related to job turnover but not to attrition from the profession.

Only about 15% of the variance in intention to leave the profession could be explained by the personal and individual characteristics included in the model (including age, gender, and career path), as well as the perception of professional demands, indicating that essential predictors were not considered in this study. Contextual factors such as student composition, school location or general workplace atmosphere may explain further variance (see Borman and Dowling, 2008 for a review). In a recent study based on the job demands resource model (Demerouti et al., 2001), Skaalvik and Skaalvik were able to show that perceived professional demands are only indirectly related to motivation to quit, via low well-being and engagement (Skaalvik and Skaalvik, 2018). In addition to the professional demands, teacher well-being and engagement would probably also explain further variance in the intention to leave the profession.

Another factor that has to be considered is that stress and dissatisfaction are not the only reasons for teacher attrition. While so-called push factors – reasons that push the individual away from his or her field of work – are highly relevant for leaving the teacher profession (Ingersoll and Smith, 2003; Herzog et al., 2007; Borman and Dowling, 2008; Struyven and Vanthournout, 2014), pull-factors – reasons that attract the individual to another field – are important as well. Feelings of challenge when confronted with professional demands may be a push factor if the necessary resources cannot be activated to master the challenges. A teacher’s degree yields good opportunities for further professional development, e.g., in educational science or special needs education. In order to explain more variance in the intention to leave the profession, it might be beneficial to look into the question whether teachers acknowledge these professional opportunities and how they are related to teacher attrition.

### Limitations and Future Research

The present study addresses several research gaps, but has limitations that can be seen as guidelines for future research. Firstly, the present study is based on cross-sectional data that do not allow any conclusions about the direction of effects, or about the processes involved. Longitudinal studies could provide insight in the processes that lead to the intention to leave a certain workplace, to leave teaching altogether, or to stay in the teaching profession despite the presence of challenges. Including potential mediating variables such as job well-being or job engagement could help to further clarify the relationship between professional demands, stress and attrition. Secondly, to assess the intention to leave the profession, only one item was used in this study. In order to assess attrition intention more comprehensively, a multi-item scale would be favorable. Thirdly, as the variables included in the model only explained a limited amount of the variance in intention to leave the profession (15% for the total sample and only 10% for FCT), it would be interesting to investigate what other predictors are related to intention to leave the profession. We assume that workplace characteristics and pull-factors might explain further variance (Borman and Dowling, 2008; Ingersoll et al., 2014). Fourthly, in this study we have examined which professional demands are challenging for the individual teachers, but not to which degree they perceive them as relevant and whether they think they can cope. However, there is empirical evidence that job demands are only dealt with thoroughly if they are considered important and manageable, but risk to be ignored or avoided otherwise (Keller-Schneider, 2010, 2014). For future research it would be important to consider the extent to which teachers feel challenged by specific job demands in the context of their subjective relevance and manageability.

## Conclusion

In sum, the study shows that SCT are more intent to stay in the teaching profession than FCT, but experience as many challenges regarding most professional demands. One exception are professional demands that are related to student learning and assessment, where SCT feel less challenged. These group differences seem to be attributable mainly to the higher proportion of male teacher among SCT, as well as to their higher general self-efficacy beliefs. Our findings indicate that persons who chose teaching as a second or third career – at least if they are well qualified as teachers – feel as prepared to cope with professional demands as traditional teachers, although not distinctly more so. However, although the previous literature suggests that SCT have additional resources compared to FCT (e.g., Tigchelaar et al., 2010; Bauer et al., 2017), SCTs’ cumulative experiences and knowledge do not diminish the challenges that they encounter when entering the teaching profession. This does not mean that SCTs’ previous professional skills are lost when they start a new career as teachers; but it indicates that the skills and knowledge gained in the past are only marginally reflected in the appraisal of professional demands as teachers. The findings also imply that SCT need teacher induction as much as any other teacher, as the professional challenges are high, especially regarding student learning and assessment.

Teachers’ career backgrounds and challenge appraisals of professional demands can only explain a small amount of variance in intentions to leave the profession again. This corroborates the previous empirical findings that challenge stressors do not have the negative consequences that are usually associated with stress, including attrition.

## Data Availability Statement

The datasets generated for this study are available on request to the corresponding author.

## Ethics Statement

Ethical review and approval was not required for the study on human participants in accordance with the local legislation and institutional requirements. The patients/participants provided their written informed consent to participate in this study.

## Author Contributions

The manuscript was written in close collaboration between the two authors. LT had the idea for the manuscript and conducted all analyses. CB wrote most of the Introduction and the Discussion section. Both authors have contributed to the manuscript to a similar amount.

## Conflict of Interest

The authors declare that the research was conducted in the absence of any commercial or financial relationships that could be construed as a potential conflict of interest.

## Footnotes

1 *Post hoc* analyses; Bonferroni adjusted for multiple comparisons.
