# Supplementary material for: Is Teaching Less Challenging for Career Switchers? First and Second Career Teachers’ Appraisal of Professional Challenges and Their Intention to Leave Teaching
Source: Front Psychol. 2020 Jan 22;10:3067. doi: 10.3389/fpsyg.2019.03067 (PMC6996299; doi:10.3389/fpsyg.2019.03067)
Supplement: Supplementary file 1 [file Table_1.docx]

Supplementary Material 1:

Items of the instruments used in the study (translated from German)

# Professional requirement scales – challenge in dealing with professional requirements (EABest-K, four factor model of professional developmental tasks; Keller-Schneider, 2014)

*Imagine your current professional situation. Rate the following demands at school on the extent how challenging they are for you. Mark the score that is most accurate (scale: 1 = little to 6 = very much).*

**Teaching to meet individual students’ needs**

Implement individualized instruction in the classroom

Structure learning tasks into different levels

Foster students individually

Assess students’ abilities to achieve

Adapt the complexity of the lesson to the students’ abilities and needs

Talk with parents about their child

Achieve confidence in dealing with parents

Lead parent-teacher conferences

**Adaptive classroom management**

Be aware of and lead classroom dynamics

Deal with different student motivation levels

Ritualize teaching procedures

Teach efficiently and avoid disturbances

Enforce the required work methods

Give clear indications and structures to the students

**Co-constructive cooperation within the school**

Find cooperation partners within the school staff

Find a position as a staff member

Represent the own viewpoints within the school staff

Develop a successful cooperation with the principle

**Professional role and identity**

Cope with own quality standards in a realistic way

Protect oneself from work overload

Find or create moments of recreation

Achieve what you want to achieve as a teacher

Act according to your own ideas of a good teacher

Care for your ongoing development

Response adequately to unexpected situations

Keller-Schneider, M. (2014 ). *Berufsanforderungen von Lehrpersonen. EABest-K, Skalen zur Erfassung der Bewältigung von Berufsanforderungen (Kurzversion von EABest).* [Professional requirements of teachers. EABest-K, Scales to measure coping with professional requirements (Short-version of EABest)]. Available: <https://phzh.ch/personen/m.keller-schneider> (accessed Mai 15, 2018).

Translation: Manuela Keller-Schneider in cooperation with Larissa Maria Troesch, Dilan Aksoy and Catherine Eve Bauer, 2019.

# General Self-Efficacy (Schwarzer and Jerusalem, 1999)

*To what extent do the following statements apply to you (scale: 1 = I absolutely do not agree to 4 = I absolutely agree)?*

If someone opposes me, I can find the means and ways to get what I want.

I can always manage to solve difficult problems if I try hard enough.

It is easy for me to stick to my aims and accomplish my goals.

Thanks to my resourcefulness, I know how to handle unforeseen situations.

I am confident that I could deal efficiently with unexpected events.

I can remain calm when facing difficulties because I can rely on my coping abilities.

I can usually handle whatever comes my way.

If I am in trouble, I can usually think of a solution.

I can solve most problems if I invest the necessary effort.

When I am confronted with a problem, I can usually find several solutions.

Schwarzer, R., and Jerusalem, M. (1999). Skalen zur Erfassung von Lehrer-und Schülermerkmalen [Scales to measure teacher and student characteristics]. *Dokumentation der psychometrischen Verfahren im Rahmen der Wissenschaftlichen Begleitung des Modellversuchs Selbstwirksame* Schulen . Berlin. Available: <http://www.psyc.de/skalendoku.pdf> (accessed December 6, 2013).

# Teacher Self-Efficacy (Schwarzer and Schmitz, 1999)

*To what extent do the following statements apply to you (scale: 1 = I absolutely do not agree to 4 = I absolutely agree)?*

I am convinced that I am able to successfully teach all relevant subject content to even the most difficult students.

I know that I can maintain a positive relationship with parents even when tensions arise.

When I try really hard, I am able to reach even the most difficult students.

I am convinced that, as time goes by, I will continue to become more and more capable of helping to address my students‘ needs.

Even if I get disrupted while teaching, I am confident that I can maintain my composure and continue to teach well.

I am confident in my ability to be responsive to my students‘ needs even if I am having a bad day.

Even if I work hard for the development of my students, I know that I can’t do much.

I am convinced that I can develop creative ways to cope with system constraints (such as budget cuts and other administrative problems) and continue to teach well.

I know that I can motivate my students to participate in innovative projects.

I know that I can carry out innovative projects even when I am opposed by sceptical colleagues.

Schwarzer, R., and Schmitz, G.S. (1999). Dokumentation der Skala Lehrer-Selbstwirksamkeit (WirkLehr) [Documentation of the teacher self-efficacy scale (WirkLehr)]. *Elektronisches Testarchiv des Zentrums für Psychologische Information und Dokumentation (ZPID)*. Available: <http://www.zpid.de/pub/tests/pt_1003tWirkLehr.pdf> *(*accessed December 6, 2013).
